# The Role of miRNA Expression Profile in Sudden Cardiac Death Cases

**DOI:** 10.3390/genes14101954

**Published:** 2023-10-17

**Authors:** Alessia Bernini Di Michele, Valerio Onofri, Mauro Pesaresi, Chiara Turchi

**Affiliations:** 1 Section of Legal Medicine, Department of Biomedical Sciences and Public Health, Polytechnic University of Marche, Via Tronto, 60126 Ancona, Italy; alessia.berninidimichele@gmail.com (A.B.D.M.); m.pesaresi@univpm.it (M.P.); 2 Legal Medicine Unit, AOU Azienda Ospedaliero Universitaria delle Marche, 60126 Ancona, Italy; valerio.onofri@ospedaliriuniti.marche.it

**Keywords:** sudden cardiac death, miRNA, circulating miRNA, exosomal miRNA, molecular autopsy, cardiac disease

## Abstract

Sudden cardiac death (SCD) is one of the leading causes of death in the world and for this reason it has attracted the attention of numerous researchers in the field of legal medicine. It is not easy to determine the cause in a SCD case and the available methods used for diagnosis cannot always give an exhaustive answer. In addition, the molecular analysis of genes does not lead to a clear conclusion, but it could be interesting to focus attention on the expression level of miRNAs, a class of non-coding RNA of about 22 nucleotides. The role of miRNAs is to regulate the gene expression through complementary binding to 3′-untraslated regions of miRNAs, leading to the inhibition of translation or to mRNA degradation. In recent years, several studies were performed with the aim of exploring the use of these molecules as biomarkers for SCD cases, and to also distinguish the causes that lead to cardiac death. In this review, we summarize experiments, evidence, and results of different studies on the implication of miRNAs in SCD cases. We discuss the different biological starting materials with their respective advantages and disadvantages, studying miRNA expression on tissue (fresh-frozen tissue and FFPE tissue), circulating cell-free miRNAs in blood of patients affected by cardiac disease at high risk of SCD, and exosomal miRNAs analyzed from serum of people who died from SCD.

## 1. Introduction: Sudden Cardiac Death

### 1.1. Definition, Etiology, and Epidemiology of Sudden Cardiac Death

Sudden cardiac death (SCD) or arrest (SCA) is described as a rapid and unexpected natural death of cardiac origin [1]. It is thought that SCD has a cardiac cause that occurs within 1 h of onset of symptoms in witnessed cases, and within 24 h of having been last seen alive and well [1,2]. To make reading easier, a glossary is provided in Abbreviation Section.

Coronary artery disease accounts for up to 80% of all SCD cases; the remaining causes that lead to SCD are cardiomyopathies and genetic channelopathies. Moreover obesity, alcoholism, and fibrosis could lead to cardiomyopathy and subsequently to a non-ischemic SCD. The types of cardiac diseases related to SCD usually depend on the individual’s age. In younger individuals, the causes that lead to SCD are mostly due to primary electric diseases and cardiomyopathies, in addition to myocarditis and coronary anomalies. Nevertheless, 50% of SCD cases throughout the fourth decade are related to coronary artery disease (CAD), especially acute coronary syndrome (ACS). In older populations, chronic structural diseases predominate either via acute coronary events or chronic coronary stenoses, valvular heart diseases, and heart failure. Over 50% of SCDs in individuals under 50 years could be caused by inherited electrical diseases or structural non-ischemic diseases [2].

In young athletes, SCD most often occurs due to hypertrophic cardiomyopathy, and in older athletes, due to coronary heart disease [3]. Interestingly, in Europe, particularly in northern Italy, arrhythmogenic right ventricular dysplasia, possibly congenital, is the predominant anatomic finding in athletes with SCD [4]. Sudden cardiac death is the most common cause of death in Western countries, and it is estimated that 4–5 million people die every year from SCD worldwide. Recent findings suggest that at least 249,538 SCD cases are expected each year in the European Union [5]. Occurrence and cause vary considerably with age of the subject, although SCD is more common in young individuals, who account for 20% of SCD cases [6].

### 1.2. Diagnostic Procedure

The diagnostic procedure for suspected SCD was published by the Association for European Cardiovascular Pathologists in 2008, and updated in 2017 and 2023 [7,8,9]. These guidelines define the role of autopsy and how SCD should be investigated. The minimum standard required for the correct appraisal of SCD in the whole population, excluding Sudden Infant Death Syndrome, is described in the procedure for routine autopsy practice, which includes not only a protocol for heart examination and histological sampling, but also a toxicological and molecular analysis [8].

The purpose of an autopsy investigation for SCD is to find out if death was caused by cardiac disease or by non-cardiac problems.

When cardiac disease is suspected, its nature should be established, i.e., to determine if it is caused by an arrhythmic mechanism (electric SCD) or by an impairment of mechanical functions of the heart and great vessels (mechanical SCD). Both disorders have the potential to affect the coronary arteries, the myocardium, the cardiac valves, the conducting system, the intra-pericardial aorta, or the pulmonary artery. The procedure for any analysis is complex and it begins with the anamnesis and, if possible, the collection of relevant information on family history, habits, on being subjected or not to medical treatment, and condition of death, together with potential reanimation events.

In addition, promising diagnostic procedures could be the imaging exams, which include postmortem endoscopy (minimally invasive postmortem), postmortem computed tomography, postmortem magnetic resonance, postmortem angiography, and postmortem multiphase computed tomography angiography [10]. An important step is also the standard histologic examination of the heart. It consists of mapping myocardium marked blocks from a representative transverse slice of the ventricles to include the free wall of the left ventricle, the ventricular septum, the free wall of the right ventricle, the right ventricular outflow tract, and one block from each atrium. Furthermore, areas with meaningful macroscopic abnormalities should be sampled. Coronary arteries can also be considered in a histological exam; more severe focal lesions should be sampled for histology in labeled blocks and stained as before [8].

Additional laboratory tests, such as toxicological exams, chemistry tests, microbiological analysis, and genetic analysis, could be necessary either during autopsy or afterwards.

A new conceptual approach for patient management that incorporates genotype-based risk stratification and mutation-specific therapies was developed through the use of molecular biology and basic electrophysiology studies [11]. When a genetic heart disease is presumed, molecular analysis is recommended. It involves the detection of mutations on DNA and RNA sequences. Mainly in younger people and in negative autopsy cases, functional reasons of death need to be considered, which include cardiac arrhythmias due to genetic causes or other cardiac disorders. So, for a complete diagnosis, a molecular genetic analysis is necessary for the detection of possible mutations [12]. As mentioned before, most of the cases of SCDs in young people are caused by inherited channelopathies, which can lead to arrhythmia syndromes, and inherited cardiomyopathies.

Channelopathies are a primary electrical disorder and the coroner cannot identify this dysfunctional disorder when carrying out an autopsy. This is classified as negative or non-conclusive autopsy, which usually occurs in the young population and accounts for 5% of all autopsies performed [13]. In SCD cases, an inherited arrhythmogenic syndrome (IAS) is highly suspected as the most plausible cause of death of cardiac origin [14].

In the forensic medicine field, a genetic analysis, the so-called “molecular autopsy”, could help in determining the cause of the SCD in young individuals in those cases that remain unexplained after a comprehensive forensic autopsy (negative autopsy) [15]. If a pathogenic or likely pathogenic variant is identified by molecular autopsy, the genetic analysis of the family members is also recommended, to lower the likelihood of additional fatalities in the remaining family members.

The concept of molecular autopsy was introduced for the first time in 2001 [16], and to date tens of genes have been identified that are associated with important causes of sudden cardiac death due to cardiomyopathy (SCD-CMP), coronary heart disease (CHD), and primary arrhythmia syndromes [17]. Recent developments, particularly with the increase in usage of next generation sequencing (NGS), have revealed hundreds of mutations in the involved genes. Ongoing research relies on guidelines of the American Heart Rhythm Society (AHRS) or the European HRS (EHRS) [18], and studying the sequence of genes associated with Long QT Syndromes 1–3 (LQTS 1–3), Brugada Syndrome (BrS), and Catecholaminergic Polymorphic Ventricular Tachycardia (CPVT). The American College of Medical Genetics and Genomics (ACMG) and the Association of Medical Pathologists (AMP) published a common declaration on the interpretation and classification of variants: pathogenic, likely pathogenic, likely benign, benign, or variants of uncertain significance [19].

The diagnosis of SCD represents a significant challenge, and notably with negative autopsy results, it requires urgent use of molecular biomarkers to understand the cause of death and to prevent severe cardiac events in the remaining family members.

## 2. Introduction: miRNA in SCD Cases

Since genome-wide association studies have found several variations in the DNA sequence involved in cardiovascular deaths (CVDs), molecular analysis has appreciably multiplied diagnostic precision in SCD cases [20,21].

Even though there are still many unknown CVD genetic risk factors, the so-called “missing hereditability” problem and the theory that epigenetics plays a role in CVD are becoming more widely accepted, recognizing that epigenetic mechanisms may partially cause a genetic risk of CVD [22]. Therefore, for each physician and pathologist, the search for reliable biomarkers of SCD is still a challenge [23].

To this end, microRNAs (hereafter miRNAs) have aroused interest due to their possible role as diagnostic biomarkers in numerous cardiovascular diseases [24,25,26]. In this review, we focus the attention on miRNAs as a group of potential new biomarkers in SCD cases.

miRNAs are a kind of non-coding RNA. Their maturation begins from a first transcribed molecule into the nucleus (pri-miRNA) and then it is processed within the cytoplasm into the active and mature miRNA of about 22 nucleotides. The main role of miRNAs is to regulate the gene expression binding complementarily the 3′-untraslated regions of mRNA. This binding can cause the inhibition of translation or it can lead to mRNA degradation [27,28]. Active miRNAs can be found intracellularly and in blood circulation, known as circulatory miRNAs. Circulatory miRNAs are present in almost all biological fluids, including blood plasma and serum, cerebrospinal fluid, saliva, urine, and breast milk. Furthermore, miRNA profiles in biological fluids can reflect the organism’s pathophysiological conditions [29].

Growing attention is being focused by researchers on the role of circulating miRNAs in cardiac disease, such as Arrhythmogenic Cardiomyopathy (AC) [30,31,32], which is one of the most common cardiac diseases that leads to SCD.

More generally, cardiac arrhythmias lead to serious dysregulation of numerous ion channels, increasing the patient’s risk of death. In this regard, it was recently observed that miRNAs have a great impact on the physiology of the heart [33]. MiRNAs regulate numerous properties of cardiac excitability, including conduction, repolarization, automaticity, Ca^2+^ handling, spatial heterogeneity, and apoptosis and fibrosis. MiRNAs can also impose their regulatory actions on cardiac excitability indirectly through targeting non-ion channel genes, such as transcription factors, which in turn regulate expression of ion channel genes [34].

Chiefly, miRNAs could prevent or trigger arrhythmias via biophysically modulating ion channels before RNA interference regulation of protein expression in diseased hearts. The control of arrhythmogenicity of the heart is aided by multiple miRNAs, and different miRNAs are involved in different types of arrhythmias under different pathological conditions of the heart [34]. Such plausible interactive networks revealed that numerous miRNAs can simultaneously target distinct components of the repolarization phase, supporting a plausible mechanism underlying co-regulation of the distinct phases of the cardiac action potential [35].

For instance, several studies have mentioned the role of miRNA 1 (miR1), which is the most predominant miRNA in the heart and controls cardiac development and function. When miR1 binds to the voltage-gated potassium channel Kir2.1, miR1 acts as a modulator of the latter, leading to a biophysical change in the function of the potassium channel. If miR1 is up-regulated, it increases arrhythmogenesis by post-transcriptionally targeting various ion channels, such as KCNJ2 and GJA1 [33].

Resuming our discussion about the general features of miRNA molecules, worth mentioning is their strong stability in extreme conditions, which suggests their involvement in cell–cell communication, and also as a response to tissue damage in the onset and development of the disease. Circulating miRNAs can be considered valid biomarkers, and several studies have evaluated circulating miRNAs differentially expressed in the heart and in plasma of patients, presenting contradictory outcomes [36,37,38,39,40].

As mentioned before, miRNAs not only have a role in the regulation of protein synthesis targeting mRNA, but they also mediate cell–cell communication. In particular, they can be released by active secretion cells within exosomes or vescicles [41]. Microvesicles are lipid vesicles in the size range of 50–1000 nm released from cell membranes, while exosomes are smaller (30–100 nm) particles released from the cell when multivesicular bodies fuse with the plasma membrane. The membrane vesicles protect the extracellular miRNAs from ribonucleases contained in serum and plasma, so miRNAs are not digested by RNase. Due to this protective action of extracellular vesicles (exosomes and microvesicles), circulating miRNAs may be considered non-invasive biomarkers in several human diseases, such as cardiovascular diseases [42,43]. It is much more challenging to understand the stableness of miRNAs in blood of individuals who have died from a sudden cardiac arrest.

Exosomal miRNAs are stronger and more precise as diagnostic biomarkers for disease-dependent pathological and physiological modifications compared to cell-free miRNAs. It is worth noting that in dead bodies, over time, postmortem changes lead to blood coagulation and the subsequent degradation process of RNA and DNA. Therefore, the postmortem analysis of exosomal miRNAs to obtain physiological information and a clinical diagnosis of the cause of death [41] could be affected by thanatological changes.

Usually when the trial is performed on a patient, it is preferable to use a non-invasive diagnostic tool, so the most accessible biological material is blood. More interestingly, in SCD cases, studies can be performed on different biological starting materials, such as fresh heart tissue or FFPE tissue.

In this review, we compare the findings of miRNA expression levels in SCD cases based on different biological starting materials, highlighting the advantages and disadvantages of having different biological tissues available. We start from miRNA expression levels in fresh cardiac tissue samples, moving on to FFPE tissue, and then focus on exosomal and circulating cell-free miRNAs in blood from SCD cases. Supplementary Table S1 reports the miRNAs mentioned in this review, together with information about miRNA types, biological materials analyzed, diagnosis, disease, and references.

### 2.1. Role of Tissue–Expressed miRNA in SCD

Yu Kakimoto and his research group from Japan carried out interesting studies into the miRNA expression profile in cardiac tissue [40,44]. In their most recent study [40], fresh cardiac tissues were collected during the autopsy from individuals who had died after heart failure and SCD, caused by cardiac hypertrophy (SCH) and compensated cardiac hypertrophy (CCH). The dead bodies were preserved frozen at 4 °C until autopsy. Cardiac tissue samples were obtained by slicing off a small piece from the left ventricular free wall, which was then immersed in liquid nitrogen and stored at −80 °C until RNA isolation. Subsequently, the authors performed RNA extraction, quantitative PCR analysis, and sequencing. The results showed that a high expression level of miR-221 in SCH patients is associated with fatal consequences, suggesting that high levels of miR-221 are probably correlated with an increased risk of SCD in patients affected by cardiac hypertrophy.

Some years earlier [44], Kakimoto’s research group focused on the stability of miRNA in postmortem formalin-fixed paraffin-embedded tissues, also measuring the expression level of miRNA in acute myocardial infarction (AMI) patients. In the forensic pathology field, AMI is considered a frequent cause of SCD due to the severe consequences on the coronary artery [45]. They compared the FFPE tissue with fresh-frozen tissue as biological starting materials for molecular autopsy. They decided to use heart tissue to analyze the postmortem expression level of miRNAs since the heart is not inclined to undergo degradation, unlike other organs, after death [46]. This study draws attention to the stability of miRNAs after death and long-term fixation, showing that miRNAs may be valid and trustworthy diagnostic biomarkers for postmortem analysis. Nineteen frozen samples were collected for the evaluation of the postmortem interval and seventeen FFPE samples were collected for the evaluation of the fixation period in formalin. The tissue collected during autopsy was formalin-fixed. This passage is necessary for histological analysis and long-lasting preservation, but it can severely damage the DNA and RNA that are present in the tissue [47].

It has been demonstrated that after 1 day of formalin fixation, the level of fragmentation and formation of cross-links into the nucleic acids of tissues reduce the PCR effectiveness and the efficiency of real-time PCR analysis [48,49]. The study points out differences in the use of fresh tissue samples and FFPE tissue samples, suggesting that the use of fresh samples can suffer from the problem of degradation due to the effects of postmortem interval (PMI), while using the FFPE tissue samples, the problem can be related to the time of the formalin-fixation period.

The results show that if the body was well preserved at 4 °C, the effect of PMI on candidate miRNAs in frozen tissues did not seem to considerably change in the first week after death. Moreover, when using the FFPE tissue samples, the effect of the formalin-fixation period on candidate gene affects detection. The longer the fixation period, the smaller the quantity of miRNAs, and each miRNA type decreased in a different time period. RT-PCR was carried out for seven miRNAs, and three other miRNAs were taken into consideration as controls for postmortem analysis. In particular, the analysis showed that miR-191 and miR-26b were well suited as controls in quantitative analysis compared to the other types of small RNA molecules because they remain stable after death and long-term fixation, and they can easily be detected. Furthermore, RT-PCR evaluates the expression of miR-1, miR-208b, and miR-499a as candidate biomarkers in FFPE tissues from AMI patients. Results report that miR-1 decreased 1.4-fold and miR-208b increased 1.2-fold in the AMI samples compared to the controls, so they cannot be considered valid biomarkers due to the minimum difference with controls and because of the limited number of samples used in this research. On the other hand, a decreased level of miR-499a was found in the AMI samples (2.1-fold compared to controls), so it can be considered a valid biomarker for AMI during postmortem examination.

As mentioned before, AMI is one of the heart conditions that could lead to SCD, and in this context, the study of an Italian research group led by Pinchi [50] investigated specific cardio-miRNAs overexpressed in FFPE heart samples from subjects deceased due to AMI or SCD, distinguishing dead AMI patients from SCD by other causes. They analyzed FFPE tissue samples in 19 cases of AMI, 25 SCDs, and 18 controls. Reverse transcription and quantitative PCR analysis were carried out for miR-1, miR-208, and miR-499. The outcomes of this study confirm the previous results obtained by Kakimoto [44], and identified miR-1, miR-499, and miR-208 as candidate SCD biomarkers with a high degree of precision to discriminate SCD cases from AMI ones, and miR-208 results to discriminate AMI from controls. In addition, Pinchi et al. found that miR-1 and miR-499 present a high accuracy in discriminating SCD from AMI (sensitivity of 0.917) and miR-208 showed the highest specificity in discriminating AMI from control cases.

Yan et al., considering the expression of other miRNAs in FFPE tissue samples, conducted a very interesting study in 2021 [51]. They investigated specific cardio-miRNAs selected on the basis of the published literature (miR-3113-5p, miR-223-3p, miR-499a-5p, and miR133a-3p) as powerful biomarkers for the diagnosis of SCD [52,53,54]. The research group selected thirty-four SCD cases, of which 18 were classified as SCD with negative autopsy and 16 were defined as SCD with positive autopsy (results from coronary atherosclerosis and gross myocardial scar).

Additionally, they selected year-matched control cases: 14 cases of fatal injury and 14 cases of carbon monoxide (CO) intoxication. Histological analyses were performed, starting from FFPE tissue from autopsies, and the same samples were also used for miRNA analysis. The purpose was to highlight the pathological changes through the use of RT-qPCR, which determines the expression level of miRNAs. Data demonstrated that miR-3113-5p, miR-223-3p, miR-499a-5p, and miR-133a-3p were useful to diagnose SCD. The study explained that these four miRNAs may be considered reliable diagnostic biomarkers for SCD, distinguishing SCD cases from other causes of death. These four miRNAs showed high expression levels in the heart tissues of SCD cases compared to controls. The ROC analysis indicated that the four miRNAs could be considered independent diagnostic markers of SCD, and combining two of these miRNAs could help to discriminate the specific causes of SCD. In conclusion, this study underlines that miR-3113-5p, miR-223-3p, miR-499a-5p, and miR-133a-3p may be considered potential candidate diagnostic biomarkers in SCD cases.

### 2.2. Role of Circulating miRNA in SCD

The specificity of miRNA expression levels in tissue and cells has been well described in other studies, including specificity for endothelial cells, neurons, hepatocytes, and cardiomyocytes [55]. Moreover, miRNA expression has been quantified in blood circulation, since it may be useful as proof of tissue modifications or systemic alterations. Previous studies have identified higher levels of circulating cell-free miRNAs 48 h after cardiac arrest in the plasma of individuals using RT-PCR [56].

In their research study, Wander et al. [57] measured 45 candidate miRNAs in plasma in individuals affected by sudden cardiac arrest due to ventricular fibrillation (VF-SCA) using RT-PCR. The trial was carried out among participants of the Cardiac Arrest Blood Study (CABS) and the different cases were divided into three groups: died in field (DF), died in hospital (DH), and survived to hospital discharge (DC).

For each case, blood samples were obtained at time of arrest, while for each control the blood samples were obtained at time of interview. The same laboratory analyzed case and control samples within 48 h of blood withdrawal. The RNA was then isolated and purified for miRNA expression measurements. Making a comparison between cases and controls, the plasma levels of several miRNAs expressed in heart, brain, liver, and other tissues were found to have higher or lower expression in sudden cardiac arrest outcomes or in individuals with coronary heart disease with a high risk of SCD. Comparing VF-SCA cases to age-, sex-, and race-matched controls, levels of 17 circulating miRNAs were higher in the sudden cardiac arrest cases. The seventeen circulating miRNAs were related to cardiomyocyte- (miR-133b, -208b, and -499-5p), skeletal muscle- (-206), hepatocyte- (-122), endothelium- (-34a), and epithelium-specific miRNAs (-205); the others were not well characterized or not tissue specific (miR-10a, -16, -183, -200a, -210, -29c, -451, and -663b). Instead, levels of three non-specific miRNAs (miR-221, -330-3p, and -9-5p) were reduced when comparing cases with controls. In a second deeper analysis, Wander et al. confirmed that levels of circulating miR-29c, -34a, -122, -145, -200a, -210, -499-5p, and -663b were higher in VF-SCA cases compared with controls, and no miRNA levels were found to be as significantly reduced in cases compared to controls. Interesting data were found about the difference between outcome groups, i.e., levels of the circulating hepatocyte-specific miR-122 and non-specific miR-205 were increased in participants who were admitted to the hospital following successful cardiac resuscitation (DC group) compared to those who died in the field (DF). Furthermore, expression levels of cardiomyocyte-specific miR-208 band -499-5p were down-regulated in the successful cardiac resuscitation group.

Comparing the DF group to the DC group, the latter had increased levels of hepatocyte-specific miR-122, miR-200a, and miR-205, and decreased levels of cardiomyocyte-specific miR-133a, -133b, -208b, and -499-5p. Moreover, comparing people who were discharged alive (DC group) with those who died in the hospital (DH group), levels of non-specific miR-9-3p and -135 were lower among the DC group.

The major limitation found in this study on cadaveric blood is that the storage conditions of biological material could interfere with the analysis. Moreover, blood must be drawn almost immediately and no later than 48 h later.

To overcome this limit, Silverman et al. [58] performed research on circulating miRNAs in blood of alive patients and researchers conducted a nested case–control study. They collected venous blood of 5956 individuals affected by CHD, tracked prospectively for SCD. If during the follow-up of cases, participants experienced the SCD event, they were matched by age, sex, race, left ventricular ejection fraction (LVEF), time of blood draw, and fasting status to two randomly chosen controls. Silverman and colleagues quantified plasma levels of eighteen candidate miRNAs and three of them were each significantly associated with a 4.8-fold increased risk of SCD (miR-150-5p, miR-29a-3p, miR-30a-5p). Although this is an interesting paper, it has several limitations, as the authors mention: first, the probability of making mistakes about death classification cannot be ignored, and secondly the study was constrained by the low number of SCD cases. Moreover, because this nested case–control study matched participants by established risk predictors of SCD, it was not possible to assess the additional discriminative ability of the miRNAs when added to established predictors of SCD. The authors also used only three prespecified miRNAs to normalize miRNA measures and, finally, the analysis did not show if the miRNAs played an active role and/or a causative role in the pathways related to SCD or if they were a sequel or epiphenomenon.

In general, we can presume that the biggest difference when performing an analysis of patients or of SCD cases is that it is easier to detect the circulating cell-free miRNAs in fresh plasma of live people, compared to SCD cases where the cell-free miRNAs go through degradation due to the effect of PMI. To overcome this limitation, in SCD cases, it is preferable to use serum for the analysis of the expression profile of exosomal miRNA because miRNAs are preserved by the lipid bilayer of vescicles and do not undergo degradation.

Starting from this assumption, it could be interesting to focus the attention on the exosomal miRNA expression profiles in the serum of SCD cadavers. A relation was observed between the onset and the progress of numerous cardiovascular diseases and the expression level of exosomal miRNAs [59]. For example, it has been reported that after an AMI, the number of extracellular vesicles in circulation increases according to the extension of the myocardial injury [60]. The injured myocardium and the mobilization of splenic monocyte are activated by the release of these vesicles from damaged endothelial cells [60]. Studying the miRNA expression profile in exosomes may be useful to monitor the physiological changes noted in subjects who died from AMI. To detect specific exosomal miRNAs in the body fluids of a cadaver, it would be appropriate to perform an autopsy diagnosis and to focus attention on the stability of exosomal miRNAs in serum.

One of the most interesting reports about the diagnostic role of miRNAs in SCD cases is Kanno’s study, which showed that if serum samples are stored at below 20 °C, the exosomes and their miRNA content remain stably preserved for at least 3 days [61]. In the same study, S. Kanno and colleagues examined the application of exosomal miRNA for autopsy diagnosis. First, they tested the stability of exosomes in the serum of cadavers in different storage conditions. Then, they analyzed the expression profile of exosomal miRNAs in the serum of four cases of acute myocardial infarction autopsy bodies and three cases of hemorrhagic shock bodies considered as controls. Lastly, they compared the different expression levels of exosomal miRNAs between AMI cases and controls, assessing their prospective role as biomarkers that determine the cause of death. Results of miRNA expression profiling performed on the serum showed that eighteen miRNAs were significantly two-fold up-regulated in AMI cases compared to controls, and sixteen miRNAs were two-fold down-regulated when compared to the CT. One of the most significantly up-regulated miRNA results, miR-126-3p, was reported to be up-regulated in serum of acute myocardial infarction cases compared to controls. Moreover, miR-145-5p, miR-143-3p, and miR-222-3p were down-regulated in AMI cases; this could explain their role in cardio-protection and the link to AMI pathogenesis when they decreased.

These findings offer a new point of view about the potential role of exosomal miRNA, both for a clinical diagnosis and in determining the cause of death, because compared to circulating cell-free miRNAs they are more stable and this feature is fundamental when considering these molecules as biomarkers [62].

It is worth noting that disease-specific miRNAs can be found not only in serum but also in other body fluids; for example, they can be found in urine or in vitreous humor exosomes, which could be potentially helpful for an autopsy diagnosis, and can also be used to establish new reliable indicators to reveal the cause of death [63].

In the last study described in this review, a comparison between circulating and tissue expressed miRNAs was addressed. In their recent study, Mildeberger et al. [64] consider the potential of using miRNAs as biomarkers in whole blood and tissue of SCD cases, with the goal of identifying biomarkers able to distinguish different categories of cardiac death.

The authors investigated three miRNAs (miRNA-1, miRNA-133a, miRNA-26a) for their diagnostic value. They examined 45 cases: 24 cases of myocardial infarction (MI) and 21 sudden unexpected deaths (SUD), of which there were 13 cases with structural abnormalities (SAs) and 8 cases without structural abnormalities. For the control group, they selected five cases of traumatic death. After a histological examination on slices of FFPE heart tissue, RNA extraction was performed on FFPE tissue samples and on whole blood samples. Quantitative analysis was then executed by two-step RT-PCR and the results were compared. The analysis of whole blood revealed that circulating miRNA-1 and miRNA-133a are up-regulated in MI cases compared to controls and compared to SUD cases, so they can be potential biomarkers to discriminate the causes of cardiac death; miRNA-26a whole blood expression is instead down-regulated in MI samples, confirming the findings of a previous study [50,65,66]. The expression levels of the same three miRNAs were also tested in FFPE tissue of the SUD cohort compared to MI cases and controls. All three miRNAs are up-regulated in the tissue of SUD cases, and miRNA-26a also showed a higher concentration. It is also interesting to focus attention on the difference between SUD structural abnormality cases and SUD without structural abnormalities; it has been demonstrated that in whole blood, circulating miRNA-1 and miRNA-133a showed a great variance in cases with SAs, but this was not the case for miRNA-26a, while in tissue all three miRNAs showed a great expression variance in the group without SAs.

The results indicate that, despite the fact that tissue is much less accessible than blood, and therefore less suited for biomarker analysis, FFPE tissue fits well in the analysis of miRNAs in this study, because in general it best reflects the current state of the human organism.

## 3. Conclusions

In this review, we have summarized experiments, evidence, and results of different studies on the implication of miRNAs in SCD cases. We compared result data from different biological starting materials showing their respective advantages and disadvantages. Moreover, we considered data on miRNA expression on tissue (fresh-frozen tissue and FFPE tissue), circulating cell-free miRNAs in whole blood, and exosomal miRNAs analyzed from serum of people who died from SCD.

The common objective of these studies is to further investigate the role of miRNAs as potential biomarkers to discriminate the causes of cardiac death. Although some studies analyzed the same miRNA types, they did not reach the same results. Some miRNAs are taken into consideration in multiple studies, such as miRNA-499a-5p, miRNA-133a, miRNA-208b, miRNA-221, and miRNA-1, and they are studied both in tissue and in blood circulation. These miRNAs have a different expression level compared to control cases, as reported in Supplementary Table S1, where we can compare the level of expression of each miRNA type. For example, miRNA-221 is found to have a higher level compared to control cases when it is studied in fresh-frozen heart tissue [43], but studying the same miRNA in circulation blood we observe the opposite result, that is, a lower level [57]. These results can be explained via reference to the paper of Mildeberger et al. [62], where it is highlighted that the use of a different biological starting materials can give different results, as occurred for miR26a. So, it is important to find a common recommendation, understanding which biological material gives more accurate results, and taking into consideration that even though tissue is much less accessible than blood, and therefore less suited for biomarker analysis, FFPE tissue best reflects the current state of the human body.

This field has to be developed because few studies have been performed and they have many limitations, such as small cohorts, which cannot represent the main population; thus, further experiments need to be carried out. In the future, it would be interesting to further explore this topic and confirm the obtained results in order to have suitable biomarkers, such as miRNAs, for postmortem differentiation of the causes of cardiac death.

## Supplementary Materials

The following supporting information can be downloaded at: https://www.mdpi.com/article/10.3390/genes14101954/s1. Supplementary Table S1. List of miRNAs considered in this paper.

## Abbreviation

ACMG	American College of Medical Genetics and Genomics
AHRS	American Heart Rhythm Society
AMI	acute myocardial infarction
AMP	Association of Medical Pathologists
ARVC	arrhythmogenic right ventricular cardiomyopathy
BrS	Brugada Syndrome
CABS	cardiac arrest blood study
CCH	compensated cardiac hypertrophy
CHD	coronary heart disease
CPVT	catecholaminergic polymorphic ventricular tachycardia
CVDs	cardiovascular deaths
DC	survived to hospital discharge
DF	died in field
DH	died in hospital
EHRS	European Heart Rhythm Society
FFPE	formaline fixed paraffin embended tissue
HCM	hypertrophic cardiomyopathy
IAS	inherited arrhythmogenic syndrome
LQTS 1–3	Long QT Syndromes 1–3
LVEF	left ventricular ejection fraction
miRNA	microRNAs
NGS	next generation sequencing
PMI	post mortem interval
SAs	structural abnormalities
SCA	sudden cardiac arrest
SCD	sudden cardiac death
SCD-CMP	sudden cardiac death due to cardiomyopathy
SCH	cardiac hypertrophy
SIDS	sudden infant death syndrome
SUD	sudden unexpected death cases
VF-SCA	sudden cardiac arrest due to ventricular fibrillation
